# A Secure, Intelligent, and Smart-Sensing Approach for Industrial System Automation and Transmission over Unsecured Wireless Networks

**DOI:** 10.3390/s16030322

**Published:** 2016-03-03

**Authors:** Aamir Shahzad, Malrey Lee, Neal Naixue Xiong, Gisung Jeong, Young-Keun Lee, Jae-Young Choi, Abdul Wheed Mahesar, Iftikhar Ahmad

**Affiliations:** 1 Center for Advanced Image and Information Technology, School of Electronics & Information Engineering, Chon Buk National University, 664-14, 1Ga, Deokjin-Dong, Jeonju 561-756, Korea; mail2aamirshahzad@gmail.com; 2 School of Information Technology, Jiangxi University of Finance and Economics, Nanchang 330013, China; neal.xiong@swosu.edu; 3 Department of Business and Computer Science, Southwestern Oklahoma State University, Oklahoma, OK 73096, USA; 4 Department of Fire Service Administration, WonKwang University, Iksan 570-749, Korea; jgskor@wku.ac.kr; 5 Department of Orthopedic Surgery, Chonbuk National University Hospital, Jeonju 561-756, Korea; trueyklee@naver.com; 6 College of Information and Communication Engineering, Sungkyunkwan University, Suwon 16419, Korea; 7 Department of Computer Science, International Islamic University Malaysia, Kuala Lumpur 53100, Malaysia; abdul.waheed@live.iium.edu.my; 8 Department of Software Engineering, College of Computer and Information Sciences, King Saud University, Riyadh 11543, Saudi Arabia; iwattoo@ksu.edu.sa

**Keywords:** supervisory control and data acquisition, industrial automation and control, remote sensing and monitoring, wireless sensor network, remote information analysis and visualization, human machine interface, protocols security, cryptography algorithms, cellular system

## Abstract

In Industrial systems, Supervisory control and data acquisition (SCADA) system, the pseudo-transport layer of the distributed network protocol (DNP3) performs the functions of the transport layer and network layer of the open systems interconnection (OSI) model. This study used a simulation design of water pumping system, in-which the network nodes are directly and wirelessly connected with sensors, and are monitored by the main controller, as part of the wireless SCADA system. This study also intends to focus on the security issues inherent in the pseudo-transport layer of the DNP3 protocol. During disassembly and reassembling processes, the pseudo-transport layer keeps track of the bytes sequence. However, no mechanism is available that can verify the message or maintain the integrity of the bytes in the bytes received/transmitted from/to the data link layer or in the send/respond from the main controller/sensors. To properly and sequentially keep track of the bytes, a mechanism is required that can perform verification while bytes are received/transmitted from/to the lower layer of the DNP3 protocol or the send/respond to/from field sensors. For security and byte verification purposes, a mechanism needs to be proposed for the pseudo-transport layer, by employing cryptography algorithm. A dynamic choice security buffer (SB) is designed and employed during the security development. To achieve the desired goals of the proposed study, a pseudo-transport layer stack model is designed using the DNP3 protocol open library and the security is deployed and tested, without changing the original design.

## 1. Introduction

In the last two decades, a number of enhancements have been made in industrial sectors such as Water, Oil, Gas, and Electric. The communication methods have been changed from standalone systems to network based systems; furthermore, to fulfill the current communication demands of industries, there is also a requirement to connect the industrial remote (geographical) located stations to one or more centralized station. To connect several remote located stations, the best, most efficient and cost effective way is to use the wireless technologies (*i.e.*, cellular, satellite, and others). Through the deployment of wireless technology, the industrial systems or the SCADA systems are able to access, monitor, and control their remotely located networked stations from a control center in minimal time; the wireless technology also overcomes the costs required with wired technology [1,2].

Human machine interface (HMI) is part of the SCADA system that provides interaction between supervisory control and data acquisition (SCADA) operators and devices. SCADA/HMIs are highly designed graphics-based interfaces in which a SCADA monitor and controlling systems are visualized; SCADA system operators use the HMI to manage the overall network structure and communication is usually displayed in the form of text (or text stream) and graphic symbols. SCADA systems are highly distributed network systems in which a number of field devices are located graphically and controlled for the main center; multimedia audio/video contents are employed at a remote location, embedded with processes, and monitored from the control center in case there is no operator available at that site [3,4]. Typically, SCADA field devices are designed for low bandwidth transmission over serial channels and the SCADA communication links and employed protocols are also limited for low bandwidth access, and are inadequate to accomplish the requirements of advance multimedia components such as audio/video. Thus, the SCADA system components need to be integrated with advanced communication networks to enable the employment or integration of advanced multimedia applications into the SCADA systems [1,4,5,6,7].

**Figure 1:**
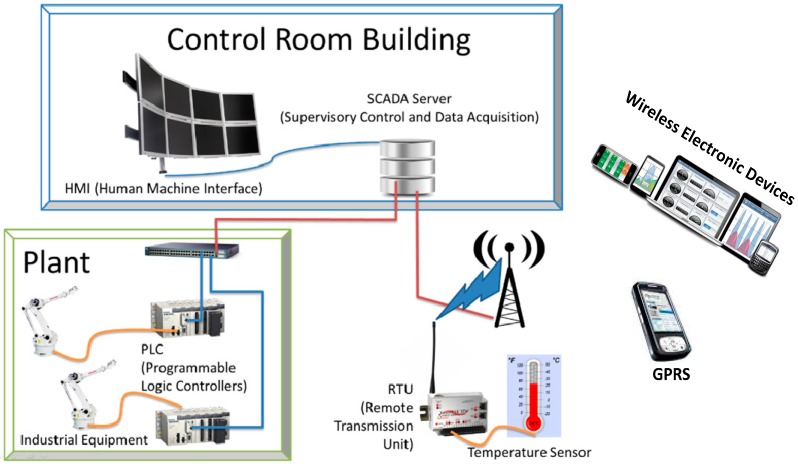
SCADA system and network components.

With the arrival of new technology, SCADA systems are also connected to numbers of advanced networks such as LAN/WAN and cellular networks; significant changes have also been observed in SCADA protocol designs and network supported specifications, which are connected to the internet for faster multimedia information delivery via non-proprietary transport control protocol (TCP). This means that the SCADA messages are constructed then passed to lower layered protocols (e.g., TCP/IP and UDP), which are usually designed to manage and meet the needs of a high bandwidth corresponding to the transmission of SCADA multimedia applications [1,8,9,10,11]. For example, the delivery time of SCADA messaging may typically be in the range of 10 ms to 100 ms, while the reliable and sequential delivery of messages (or packets) is also an important factor in the SCADA system. If we transmit the packets using SCADA serial protocols (e.g., DNP3 protocol and Modbus protocol) over Ethernet LAN using 100 Mbps transmission link, then a SCADA message (or multimedia message) can be sent in less than or within the range of 10 ms. However, converters and/or gateways such as 5201-DFNT-DNPM, Moxa NPort 6110, VLINX MODBUS, and PLX31-MBTCP-MBS are used during the transmitting of a message from SCADA serial protocols to TCP/IP protocols and vice versa. Nowadays, SCADA predominated protocols such as the DNP3 protocol and Modbus protocol are also available in TCP/IP versions as the DNP3 TCP/IP protocol and Modbus TCP/IP protocol, respectively; the frames are constructed according to protocol specifications and directly encapsulated into TCP/IP packets to ensure reliable transmission over LAN/WAN [1,11,12,13]. Moreover, the network architecture of the SCADA system is illustrated in Figure 1.

In SCADA system, DNP3 protocol has been considered as an major protocol due to its functionalities and reliable communication over the physical channels, but at the same time it has several security issues while travelling over the open networks and/or Internet [11,13]. As a consequence, the proposed study focuses on the DNP3 pseudo-transport layer security issues that most often occur during the transmission of fragments and a corresponding security mechanism or hashing function is deployed as a strong security wall that provides protection against adversaries (*i.e.*, integrity attacks); however, this development is also able to test the other cryptography algorithms according to security demands. In addition, attack scenarios are defined in which attackers gain access to the fragments, user defined attacks are launched by employing various built-in tools for performance evaluation purposes, formal proofs are employed for validation purposes, and approximate communication is visualized as part of the multimedia technology.

The remainder of this research paper is organized as follows. Section 2 reviews the related works of study. Simulation Design and Environment is explained in Section 3, Section 4 describes the Pseudo-Transport layer message structure, Section 5 describes the Payload Design and Security Development, and Section 6 explains the Algorithm. In Section 7, Attacking Scenarios are defined, while the Setup and Abnormal Communication, as well as Measurement and Discussion are explained in Section 8 and Section 9. Multimedia Contexts are highlighted in Section 10. The significance of the study is discussed in Section 11 and Section 12 provides the conclusion and suggestions for future research.

## 2. Related Work

SCADA system security issues [14,15,16,17,18,19] have been considered as the most prominent and important counter measures of communication [11,12,13]. Therefore, an evaluated potential method is proposed that would be significant to fight against SCADA security challenges; however, security enhancement is limited to specified goals (or security goals). Typically, SCADA system networks and their components are distributed in various locations including in one specific place, in many cities in a country, and around the world. To connect the several networks points, SCADA has been employed in various wired/wireless communication media and the transmission can be accessed over modern technology platforms such as cellular phones using 2G, 3G, 4G, and general packet radio service (GPRS) [20,21,22]. However, overall SCADA communication is carried out by non-proprietary protocols which are ranked above the SCADA proprietary protocols [11,12,13,14].

**Figure 2:**
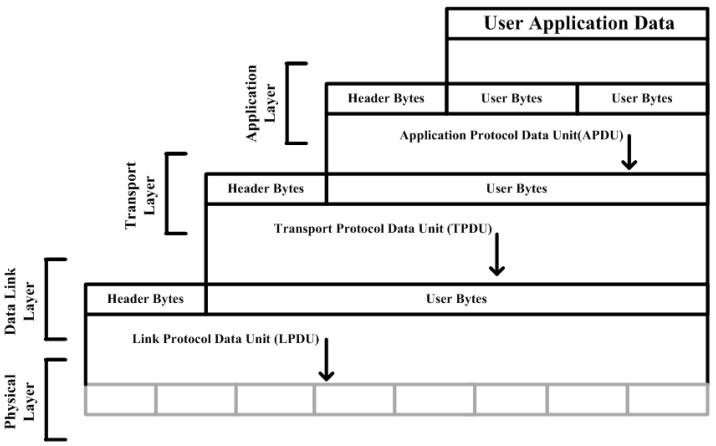
DNP3 protocol model.

The larger SCADA system defines the communication structure between the master terminal unit (MTU) and the remote terminal unit (RTU) or/and RTU and MTU. Each station is identified as a master or client/slave station in the SCADA network. However, in a SCADA hierarchical structure, some field devices perform the function of master and slave together. Two terms are defined within the data link layer such as balanced and unbalanced communication. In the application layer, the application protocol control information (APCI) defines data/message that is requested/responded; and response header differs by two additional bytes designated as internal indications (IIN). In the case of an unsolicited response, message is received from the terminal station to the master station and the master station responds to the terminal station. Therefore, different forms of header are added during message construction in the application layer, while the data link layer link protocol data unit (LPDU) bytes remain unchanged in either the message sent from the master station or the terminal station [20,21]. In an unbalanced system, only the master station is able to send the request and will respond according to the request slave station. This means that the master station works as a primary station and other stations work as terminals in an unbalanced system. Whenever the master station sends a request, the substation will then be able to send a response to the master station. However, in a balanced system, each station in the SCADA hierarchical structure acts as a master or slave at the same time. To distinguish between the master and the outstation in the balance system, a direction bit or DIR is set within the message from the master station to the terminal station or from one station to another station. Therefore, any station can initialize or send a request to other stations in the SCADA network. As part of the link layer, a cyclic redundancy check (CRC) is employed which performs the function of detecting errors in the transmission, while the detection mechanism is limited for information authentication and authorization [20,21]. Figure 2 illustrates the DNP3 protocol model and data link layer design [20].

Taxonomy of DNP3 protocol attacks is developed, in which attacks are categorized into three main groups: (i) DNP3 specifications attacks; (ii) DNP3 vendor based attacks; and (iii) DNP3 underlying infrastructure attacks [13]. The DNP3 specifications attacks are more prominent and harmful among the other attack groups; The SCADA system is targeted, which then suffers, and in this case, transmission is carried out by the DNP3 protocol [12,13]. The initial DNP3 protocol was designed without considering security; on the other hand, the DNP3 protocol resides in non-proprietary protocols (*i.e.*, TCP and UDP) for the purpose of information delivery on the internet [12,13,18]. As analyzed, three issues of interception, interruption, and modification always interact with the SCADA system and/or its component paths, including the main controller, outstation (or sub-controller), and communication network [11,12,13]. Typically, the DNP3 protocol design does not deploy the potential security mechanisms such as authentication, encryption, and authorization. Due to security limitations, outside attackers can easily interrupt the DNP3 transmission, or directly target the DNP3 layers including the application layer, the pseudo-transport layer, and the data link layer; and the configured DNP3 nodes are also not able to analyze that the incoming message, and its contents are valid, or have not been changed during transmission [11,13,14]. The attack taxonomies for the DNP3 pseudo-transport layer and data link layer are depicted in Table 1 and Table 2 [13].

DNP3 protocol layers such as the application layer and link layers are considered more vulnerable to security threats than the pseudo-transport layer. This is because the pseudo-transport layer provides fewer functionalities than the other layers of the DNP3 protocol; therefore, a limited number of attacks are linked with the pseudo-transport layer [11,13,23,24,25]. However, two potential attacks account for the pseudo-transport layer: first frame (FIR) and final frame (FIN) flags interruption and sequence number modification [11,12,13]. In the DNP3 original design, there is no defined mechanism that detects abnormal entities in the transmission; therefore, DNP3 devices (or nodes) are unaware in cases where unauthorized entities are successful in transmission by attacks such as interruption, modification, and fake reply [11,12,13,16,17,18,19,20,21,22,23,24,25]. However, the major explained pseudo-transport layer attacks fall under the category of integrity attacks and should be resolved by employing cryptography based integrity functions.

In [26,27,28,29], cryptography based end-to-end security mechanisms are used for SCADA systems, and various cryptography algorithms such as symmetric (*i.e.*, AES and DES), asymmetric (*i.e.*, RSA, Diffie-Hellman, and DSS), and hashing (*i.e.*, MD5 and SHA2) algorithms are deployed to secure the SCADA communication from networks adversaries such as message sniffers, man-in-the-middle attackers, eavesdroppers and password crackers, data interruption, and modification attackers, and others. As a consequence, cryptography based developments are considered more reliable and secure developments for SCADA systems [11,25,26,30,31,32,33,34,35,36,37,38,39,40]. In symmetric encryption, while the desired message is encrypted, this does not ensure that the message contents are not modified during transmission because a single secret can be shared between the sender and the receiver. Therefore, public key encryptions are considered to be better approaches than symmetric encryptions; in addition, a non-repudiation security service should be achieved while employing the public key encryption with hashing function, or by employing the digital signature technique [11,25]. In [25], an end-to-end security solution was implemented in the transmission of the SCADA system. The SCADA nodes such as the master terminal unit (MTU) and the remote terminal unit (RTU) were installed with DNP3 protocol, and were configured in the SCADA testbed setup. In the testbed, communication is initiated from the MTU and the desired message is treated with a hash algorithm and public key encryption before transmitting to the destination. The message hash digest is computed by employing a hashing function and the computed hash value is then encrypted with a private key for the received message (or RTU). The message does not encrypt itself, and this minimizes the computation time of the encryption process. At the RTU side, the MTU public and RTU private keys are deployed and the MTU/RTU hash values are compared to verify the message contents. In the testbed, each node is installed with a snort tool that monitors the traffic and a snort analyzer is used to detect the intrusions and generate corresponding alerts during communication between the MTU and RTU and vice versa [11,25,37,40].

## 3. Simulation Design and Environment

**Figure 3:**
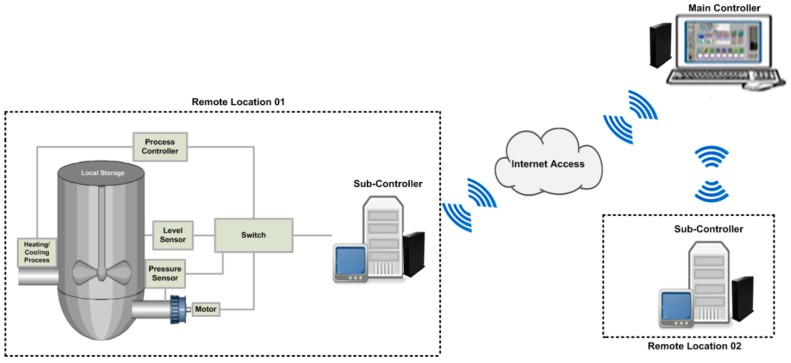
Simulation design and environment.

To measure the desired goals of current study, a simulation environment is designed for water pumping system as a part of wireless SCADA system. In wireless SCADA system, the field devices’ (or field sensors) are configured and directly connected with the sub-controllers, which are designated to carry the real time information from the sensors, or to monitor the real time information, as required by the main controller. The main controller is superior in the whole system design and network setup and is authorized to send the commands to the field sensors through the sub-controller(s). In water pumping system, as shown in Figure 3, only its two functional parts are considered: pumping for the cooler and pumping for the heater, the heating/cooling points are measured in-accordance to the normal set points that added at the time of configuration; and alarms are generated in-case the abnormal points or critical points will be measured from the field sensors. In wireless SCADA systems, each network node, such as sub-controller and main controller, is installed and configured using of DNP3 protocol as a part of SCADA system. Each time communication has occurrs between the nodes, the message is generated by deploying of DNP3 specified message structure and transmitted between the networked nodes, through employment of WAP (Wireless Application Protocol), the SCADA/DNP3 system would able to made the connection and to communicate wirelessly, to its remote located terminals (or remote field devices). In conclusion, the proposed study uses the SCADA/DNP3 protocol for messaging, the TCP/IP protocols to communicate over the Internet, and WAP for wireless communication; and moreover to secure the communication of wireless SCADA system, the cryptography hashing algorithm is deployed and tested at the pseudo-transport layer of SCADA/DNP3 protocol. The details for: message design, security design, security implementation, and security testing, are described in the below sections, of this study.

## 4. DNP3 Pseudo-Transport Layer

The pseudo-transport layer is the second layer of DNP3 after the application layer. The pseudo-transport layer takes the application protocol data units (APDUs) from the application layer of the DNP3 protocol and the upper layer bytes are treated as a transport service data unit (TSDU) or user bytes in the lower layer (or in the pseudo-transport layer). The main function performed by the transport layer is the disassembling and reassembling of bytes. The disassembling and reassembling processes allow a larger block of user data from the application layer to be handled easily by a data link layer [20]. In this research, transport protocol data units (TPDUs) are constructed as part of the DNP3 transport layer. Subsequently, control should be passed to the security development process where the hash function is applied using the SHA-2 hashing algorithm, as part of the cryptography mechanism.

### Message Structure

**Figure 4:**
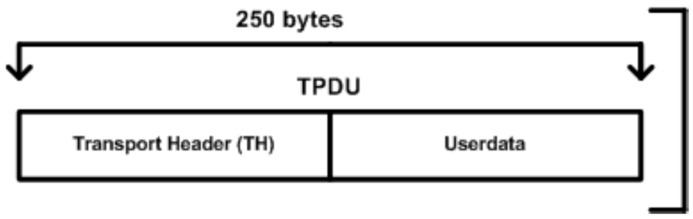
Single TPDU block.

**Figure 5:**
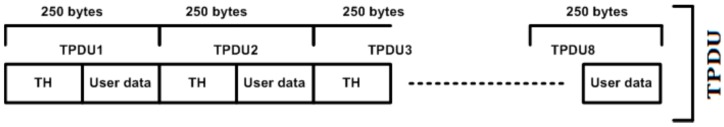
Multiple TPDU blocks.

The pseudo-transport layer breaks the TSDU into a number of units called transport protocol data unit (TPDUs) and each TPDU is made up of 250 bytes including 1 header byte. In Figure 4, 249 bytes are added with 1 byte of transport header (TH) information; this TH was originally named transport protocol control information (TPCI). In the case where a complete payload (or 2048 bytes information) has been received from the upper layer, the APDUs are then generated according to the payload size. In Figure 5, a total of eight TPDUs are generated and the remaining 56 bytes of the Application Protocol Data Unit (APDU) or 32 bytes of the cyclic redundancy check (CRC) from Link Protocol Data Unit (LPDU) would be employed for especial purposes. The size of each TPDU is fixed to 250 bytes because TPDU block could easily fit within a frame of the data link layer. This study made an alignment of the APDU which could be easily assembled within one segment (or TPDU) of the pseudo-transport layer.

**Figure 6:**
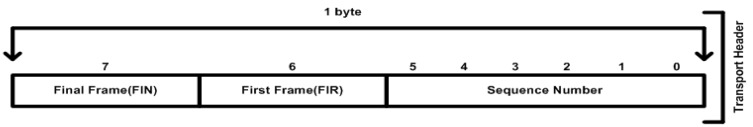
Transport header field structure.

The transport header is composed of three fields: FIR, FIN, and sequence number. Each TPDU is 250 bytes in length, which easily fits into the data link layer frame, called FT3. In Figure 6, the TH contains one byte of information and each bit has a specific function. The last two bits define the start and end of the TPDU sequence and the remaining six bits define the sequence counter.

## 5. Payload Design and Security Development Using Hashing

Similar to other SCADA protocols, the initial design of the DNP3 protocol was also limited in terms of security, or the security design was associated with the physical parts of the system [11,12,13,14]. To fulfill the requirements of industrial processes and automations, SCADA systems are connected to almost all modern networks [20,21,22]. To minimize the security falls that have been associated with communication of the SCADA system, several studies [25,26,27,28,29,30,31,32] have been conducted that provide node-to-node security protection against various vulnerabilities [12,13,14,41,42,43].The DNP3 application layer and data link layer security have been analyzed and various cryptography techniques have been suggested to enhance the security of these layers, but are still under development [11,22,23]. As a consequence [12,13,22,23], security issues have seldom been considered for the pseudo-transport layer. The current research therefore emphasizes the pseudo-transport layer security issues and deploys a cryptography mechanism as the best approach to significantly enhance the security of this layer.

While the security development at the pseudo-transport layer is simple and straightforward, fulfilling the requirements of the pseudo-transport layer design, or its functional specifications, is more complex. However, we employed the C# tool to design and construct the transport layer bytes and employed the security development process using the SHA-2 hashing function. The entire development is also validated through proofs and evaluated through computed results.

This section is divided into three Sub-Sections: Section 5.1 Payload Design and Computation; Section 5.2 Security implementation; and Section 5.3 Proof of development. In Section 5.1, the transport layer payload is computed, and further described in Section 5.2 for the purposes of security computation. Section 5.3 demonstrates the proof of development from Section 5.1 and Section 5.2.

### 5.1. Payload Design and Computation

**Figure 7:**
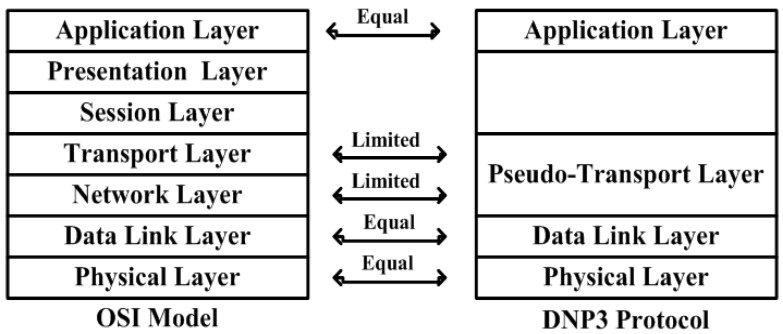
Interconnection between OSI model and DNP3 protocol.

In the DNP3 stack, the pseudo-transport layer takes the APDU as the user bytes from the application layer, and assembles the upcoming bytes into TSDU (bytes). In the reassembling process, the transport layer receives each TPDU (bytes) from the data link layer, and the TH is then stripped off and the TSDU bytes are recreated (or reformed) from the tripping process of TPCI. The pseudo-transport layer is also responsible for ensuring the sequence of TPDUs during the TSDU reassembling process. Due to the disassembling/reassembling process of the pseudo-transport layer, the data link layer is able to handle the bulk of the data, but the functionality is finite in the transport layer of the open systems interconnection (OSI) model (as illustrated in Figure 7) [20].

**Figure 8:**
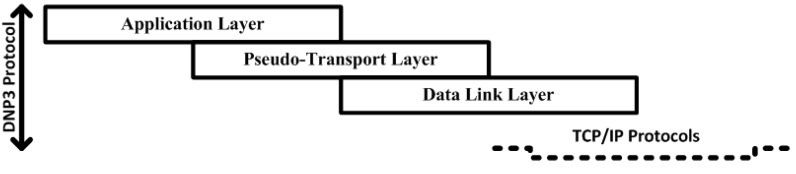
Logical interrelation and communication flow.

The DNP3 protocol is a proprietary protocol and its design is limited for advanced IP based client/server applications; therefore, TCP/IP protocols are employed instead of the DNP3 physical layer, to communicate over networks such as LAN/WAN and over the internet. Figure 8 shows the pseudo-transport layer interrelation and flow of communication.

As described above, the overall development has been made in the C# platform and in a few available implicit code libraries. Examples are employed as references [44], with user defined codes to validate the approximate and best development, according to the best of our knowledge. The following definitions demonstrated the pseudo-transport layer payload design, and its operations.

**Definition 1 (Bytes Assembling):**  The number of user bytes “B” is received by the interaction of variable “Q” and “$f_{Q}$” is an explicit dual non-linear function which assembles the upper layer bytes “$\prime B_{APDU}$” with the lower layer bytes “$B_{TSDU}$” and vice versa by the interaction of “Q”. However, since “B” is limited, an integer “${\mathbb{Z}}^{*}$” (*i.e.*, not negative integer) exists if “B” defines the limit as lim←k, such that,

$$\Leftrightarrow B_{APDU}\propto B_{TSDU}\Rightarrow f_{Q}:\;B_{APDU}⟼⟻B_{TSDU}$$

**Definition 2 (Bytes Dissembling):**  Assume b∈B, where “b” refers to the fixed/non-fixed number of user bytes during the disassembling process of “$B_{TSDU}$”. In the case where i=0 or $i_{0}$ is manipulated, then b∈B∈∅, such that,

$$B_{TSDU}\;\Rightarrow$$

$$\overset{lim\leftarrow k}{\underset{B(b,k)}{\sum }}b_{i\in \left(\emptyset ,n\right)},\;i=0,1,2,3,\ldots \;\ldots ,\;n-1,\;n,b\in B\leq lim$$

**Definition 3 (Payload):**  “α” is a variable that counts the number of bytes “b”, and the explicit user function “$f_{\alpha }$” is employed to manipulate the transport layer (TL) user bytes $Q_{b\leq \left(\lim,\emptyset \right)}^{TL}$ corresponding to the disassembling process, with header (h) functional bytes $Q_{h,h\neq \emptyset }^{TL}$, where $Q_{\left(h,b\right)}^{TL}\leq \text{lim}$.

### 5.2. Bytes Alignment and Security Computational Bytes

During the payload design and computation, a keyword “limit” (lim) is defined, the purpose of which is twofold: (1) limit the number of bytes in each TSDU; and (2) limit the number of bytes in each TPDU. However, the size of TSDU is directly proportional to the size of APDU, but the size of each TPDU is limited to 249 bytes, plus 1 byte of header [20,21]. In this study, we limited the upper bytes (or APDU) size to 1992 bytes in both cases: request and response payload. This would further align with the TPDUs. For example, if we define the size of APDU as 1992 bytes, then eight equal TPDUs are created, as an addition to the transport protocol control information (APCI). This would also significantly protect information from non-legitimate users; fixed sized data is transmitted rather than variable size data.

For the alignment process of APDU and the fixing of TPDUs, the remaining 56 bytes are employed to keep track of security development and to protect sensitive information from unauthorized users. Hence, all remaining 56 bytes are not employed in this development, but are utilized and considered for other parts of the DNP3 protocol security enhancement purposes [37]. Some functions are deployed by employing the bytes from the total of 56 bytes, while the remaining functions are padded with zeros to be un-padded later for future developments. The functions details are as follows.

- Payload Counter (Two Bytes): Payload (or TPDU) is created, and 250 bytes are counted in the payload counter. In the case where minimal bytes are defined, the remaining bytes are padded to protect the payload from data modification and reply attacks.
- Hash Sequence Counter (One Byte): In the case where the number of TPDUs is defined by a single TSDU, the hashing sequence is counted in the range of 0–63, and should be recycled as 63–0 on the remote side. Two bits are used that designate the first and last hashing sequence in the defined range.
- Security Method (One Byte): In the proposed study, SHA-2 hashing is deployed to protect the sensitive information of the transport layer against integrity attacks. However, this development is also able to test other algorithms such as secret key and public key algorithms. In this case, if multiple algorithms have been deployed, the dynamic selection is made by this functional field.
- Padding Counter (Two Bytes): Initially, two bytes are defined that accumulate the number of padding bytes in the entire development. The size would be changed as required by this functional field by allocating bytes from dynamic storage (DS).
- Acknowledge No (Two Bytes): Acknowledge flags are set at both sides of the communication. Therefore, acknowledgement is required at both sides followed by the acknowledgement number.
- Useful Contents (One Byte): Typically, the payload contents are verified corresponding to the content list before being transmitted to the networks (or remote site).
- Dynamic Storage (10–46 Bytes): Bytes are dynamically allocated to other fields, if required; or these bytes are reserved for future development.

### 5.3. Security Implementation

In the DNP3 protocol, the pseudo-transport layer performs a limited functionality of the transport layer and data link layer of the OSI model. As described, the functionality is fairly limited; therefore, the vulnerabilities are also limited, or a limited number of attacks are linked with the pseudo-transport layer [12,13]. In [13,18], three commonly potential attacks including Interruption, Modification, and Fabrication, with 32 instances, are counted against the pseudo-transport layer in terms of security, two of which are directly linked with the TPDU flags and their sequence in the DNP3 transmission. However, data modification, fake messaging, and byte interruption are considered as part of the current research. The SHA-2 hashing algorithm is deployed, and is considered in order to enhance the security of the pseudo-transport layer as part of the DNP3 protocol; this development is also able to test the other security algorithms [11,25].

In security implementation, the remote terminal station (RTU) is responsible for generating and sending responses according to the main controller request. The proposed work is based on a simulated environment and the scope is limited to pseudo-transport layer security; therefore, we do not give a detailed explanation of the phenomenon of the client/server architecture. The following steps are followed to deploy the SHA-2 algorithm, and to enhance the security of the pseudo-transport layer, while Table 3 summarizes the notations that are employed in the development.

- $Q_{h,h\neq \emptyset }^{TL},Q_{b\leq \left(\lim,\emptyset \right)}^{TL}⟹Q_{\left(h,b\right)}^{TL}$ is the transport layer payload that is being manipulated by security function $H_{digest(s,Q)}^{TL}$ using SHA-2 algorithm. The maximum size of each $Q_{\left(h,b\right)}^{TL}$ is 250 bytes, if a number of $Q_{\left(h,b\right)}^{TL}$ are created then the hash sequence is counted to keep the track of each $Q_{\left(h,b\right)}^{TL}$. The original payload $Sender\left(s\right):\;Q_{\left(s,h,b\right)}^{TL}$ and computed security function $Hash\left(H\right):\;H_{digest(s,Q)}^{TL}$ are transmitted, while the parameter, which designates the sender information, is added.
- Upon receiving at the other side, the receive hash digest $H_{digest(R,Q)}^{TL}$ is computed based on the original payload $Sender\left(s\right):\;Q_{\left(s,h,b\right)}^{TL}$ and compared with $H_{digest(s,Q)}^{TL}$. As a consequence, if $H_{digest(R,Q)}^{TL}=H_{digest(s,Q)}^{TL}$ then the payload would be accepted; otherwise, it is rejected in the case of $H_{digest(R,Q)}^{TL}\neq H_{digest(s,Q)}^{TL}$.

**Figure 9:**
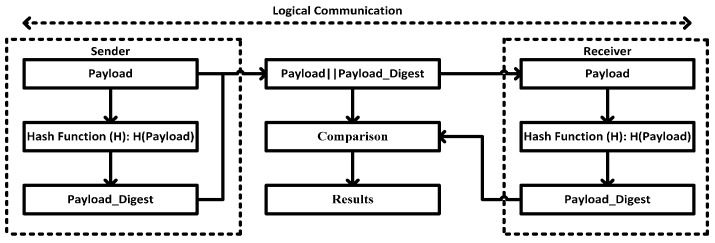
Security development Using hashing function.

In security development (or in Figure 9), the number of integrity attacks such as data modification, data detection, and data reply could be verified in the transmission and this would also be concluded in the security (or lack of security) of the pseudo-transport layer. More detail is described in Algorithm 1, in Section 6.

## 6. Algorithm: Pseudo Code Transport Layer Message Construction with Security Design

**  Algorithm 1: Transport Layer Security.**  Input: The input of Upper Layer Bytes.  Output: Transport protocol data unit (TPDU), Hash digest.  The input of “n” bytes is received from upper layer. In pseudo-transport layer, these bytes are assembled as transport service data unit (TSDU) .Hence, the bytes are limited are upper layer therefore, we defined TSDU corresponding to APDU.  TranLayer Header TH = 1 byte, TranLayerUserdate UD = 249 byte, TranLayer TPDU = 250 byte;  Bytes TSDU [ ] = [ ]; Bytes H [ ] = [ ]; Bytes UD [ ] = [ ]; Bytes TPDU [ ] = [ ];  2.TranTPDU ( ){ Bytes TPDU [ ] = [H [ ] = [ ], UD [ ] = [ ]]; Bytes H [ ] = [Sequence No., FIR, FIN];} 3.TranTSDU ( ){ Bytes TSDU [ ] = [TPDU1 [ ], TPDU2 [ ], TPDU2 [ ] ,………. , TPDUn [ ] ];Where n = 1,2,3,……….., n and maximum size of each TSDU is equal to APDU size.If TSDU [ ] = APDU [ ]Print (“Process transport layer Information”);else if TSDU [ ] = APCI [ ] Print (“Process transport layer Information corresponding to application header or APCI ”); Else Print (Unknown Communication); 4.Cryptography ( ){ RequestMessage M, j, Hash Digest HD ;Hash ( ) //Sender function{ Total Payload = Hash(M)$\;i={\text{TPDU}}_{0}+{\text{TPDU}}_{1}+{\text{TPDU}}_{2}+\ldots +{\text{TPDU}}_{n-1}+{\text{TPDU}}_{n})$, i = 1, 2, 3 ….limit. Here, $i={\text{TPDU}}_{0}$ is defined, if only header bytes are manipulated and limit shows the maximum value, or maximum value of “n”.Bytes M[ ] = [ ];For ($i={\text{TPDU}}_{1}$; i <= ${\text{TPDU}}_{n}$; i++);Print (“Add in to Hashing Buffer” +M[ ]);$\text{ Sender}:\text{Hashing}\Rightarrow \text{SHA}2[{\left(\text{TPDU}\right)}_{i}]={\left(\text{TPDU}\right)}_{i}\_\_\text{Digest}$ 5.Hash ( ) //Receiver function{ Total Payload = Hash(M)Bytes M[ ] =[ ];For ($i={\text{TPDU}}_{n}$; i <= ${\text{TPDU}}_{1}$; i--); Print (“Add in to Hashing Buffer” +M[ ]);$\text{ Receiver}:\text{Hashing}\Rightarrow \text{SHA}2[{\left(\text{TPDU}\right)}_{i}]={\left(\text{TPDU}\right)}_{i}\_\_\text{Digest}$Comparison: $\text{Receiver}:{\left(\text{TPDU}\right)}_{i}\_\_\text{Digest}=\neq \text{Sender}:{\left(\text{TPDU}\right)}_{i}\_\_\text{Digest}$ 6.Conclusion: At sender side, hash function is deployed on TPDU and computed and compared at remote side (or at receiver side) to verify the integrity of payload (or APDU).

## 7. Attacking Scenarios

In the pseudo-transport layer header or transport protocol control information (TPCI), one byte is designed to represent the header information, six bits define the sequence number counter, and the remaining two bits are employed to designate the FIR and FIN frames of APDU (or fragment) [13,20]. In the transmission, the number of frames of a payload are sent and counted in sequence, where the FIR and FIN terms define the special meaning in the processing of the payload. In the case where the payload is transmitted with the FIR indication flag set, all the existing fragments (or partially-completed fragments) are then wasted, and are no longer considered. In some scenarios, the sensitive information of the pseudo-transport layer is interrupted.

- In the reassembling process, the original payloads are disrupted; if a newer payload enters with the FIR flag set, the fragmented payload transmission subsequently starts.
- The numbers of payload are transmitted and counted in the sequence counter while the sequence should be recycled at the remote side. An interruption is created during the manipulation of the incomplete (or partially completed) payload if the new payload is entered with the FIN indication flag set; as a consequence, the assembling process is closed, as it is untimely.
- The APCI information is sensitive, and needs to be protected from unauthorized entities. The adversary has many chances to delete the payload information during transmission. The attacker uses sensitive information by using various capturing tools [11] and deletes/modifies the flags set such as FIR and FIN, and at the remote side, the receiver assumes that the payload originates from a secure source.
- In APCI, 6 bits are occupied by a sequence number (field) which ensures the transmission of a fragmented payload (APDU) in a sequence order. Each time, the fragment is created and transmitted, and the corresponding number is added to the sequence counter; thus, the transmitted and transmitting fragmented payloads are recorded with a unique sequence number. However, an attacker could have many chances to change the fragment sequence, monitor the traffic, and capture the fragments. Using a sequence number, an attacker employs various inject tools to change the sequence counter value and to inject a new fabricated fragment instead of an original fragmented payload [11]. As a consequence, there are many scenarios in which the sensitive information of the pseudo-transport layer can suffer from internal/external adversaries [12,13,18].

Security approaches [26,27,28,29] have been proposed to hide sensitive information from attackers [1,2,3,4,5,6,7,8,9,10,11,12,13,18], but these security approaches are limited in terms of specification design, protocol dependencies, and transmission requirements [11,25]. To hide the information, cryptography approaches are considered as the best solutions for system security [26,37]; in a few cases, the encrypted information cannot be satisfied at the remote side, especially during decryption of the header [11,37,43]; therefore, the best solution is to encrypt the user bytes, excluding the header bytes [43]. On the other hand, if header information is not secured, there is a chance an adversary [12,13] modifies the header with false information while replying to the message. Therefore, in this research paper, a hashing algorithm was employed that generates a fixed size security code and travels along the original payload that keeps the receiver aware of unauthorized opponents. In the following section, attacking tools are employed to interrupt the normal flow of the pseudo-transport layer as part of the DNP3 protocol, and the corresponding observed measurements are discussed.

## 8. Setup and Abnormal Communication

In a SCADA wireless network setup, the number of nodes is configured to exchange information with the main controller, although the total number of SCADA nodes is not discussed here because of unicasting communication; the system is designed according to the terminologies of an unbalanced system in which only the main controller is authorized to initial communication with the remote controller(s) [20]. However, the terms such as balanced and unbalanced, which are defined at the data link layer, are not part of the pseudo-transport layer. Therefore, this study does not specifically emphasize these terms, but we conclude that, to the best of our knowledge, the unbalanced system is more appropriate than the balanced system for this study.

To interrupt the logical normal flow of the pseudo-transport layer, predominated attacking tools such as airpwn, file2air with wireshark, and injection tools, are used which perform traffic monitoring and frames (or fragments) captured as an attacker of the system [11]. However, security development is limited to an integrity security service; thus, the attacks such as fragment injection, payload replay, and payload deletion are considered as corresponding to the proposed security implementation.

## 9. Measurement and Discussion

DNP3 protocol unitization has been massively increasing (*i.e.*, 70%) in SCADA systems [13]. Due to the lack of security precautions in the initial design of the DNP3 protocol, several potential adversaries take advantage of the DNP3 protocol’s vulnerable platform [11,13]. The current study employs a hashing function to enable awareness between the SCADA and DNP3 nodes, if transmission is interrupted by network adversaries. This research paper also deals with various developments of multimedia based security followed by communication requirements, although the study scope is limited to the pseudo-transport layer, as a layer of the DNP3 protocol.

**Figure 10:**
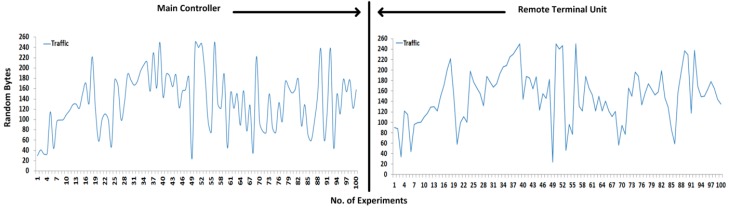
Traffic: (**left**): Main Controller; (**right**) Remote Terminal Unit.

To compute the performance measurements, random size fragment payloads are generated several times and transmitted between the main controller and the remote controller and vice versa; however, each fragment is limited to 1–250 bytes in length. In the case where no TSDU bytes are assembled from the upper layer, only the TPCI is transmitted with the computed hashing code. In the transmission, each fragment hash digest is calculated before transmitting to the remote side; the fixed hash code (or digest) travels along the original payload and is again computed at the receiver side to verify the contents of the payload. Of all the experiments, 200 are selected as the best experiments according to the best of our knowledge, and further performances such as attacks detection and security assessment, are also based on these selected experiments. Figure 10 shows the 200 successful experiments that are tested with random size payloads (or segments) and received at the remote side, whereby the first half of all the experiments are designated for sending to the payload and the remaining half are designated for the response payload. Each half is separated by a line.

**Figure 11:**
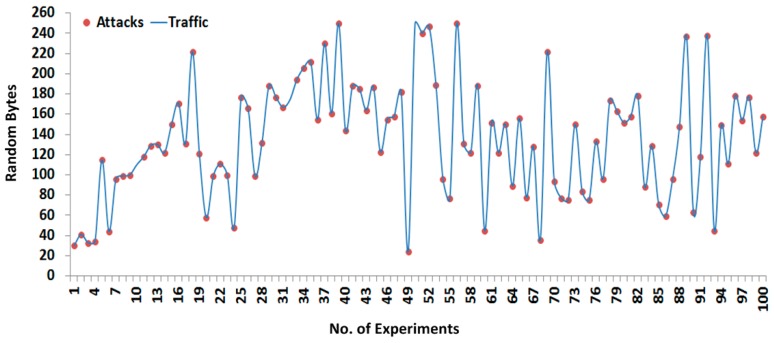
Attacks: Main controller traffic.

**Figure 12:**
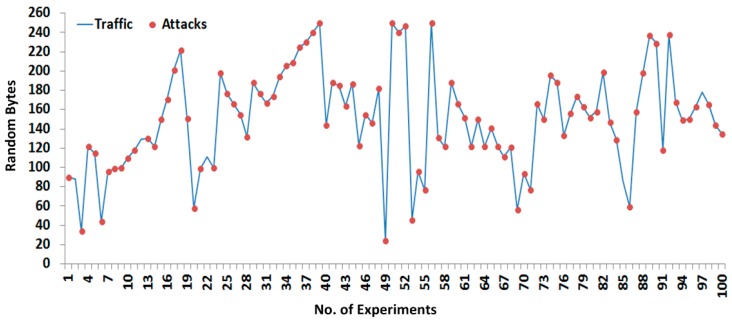
Attacks: Remote terminal unit traffic.

Figure 11 and Figure 12 show the 200 successful attacks experiments that are tested with random size payloads (or segments), whereby 100 successful experiments are designated for sending to the payload and the remaining 100 as shown in Figure 12 are designated for the response payload.

To evaluate the performances, preliminary packet analyzer tools such as wireshark, dSniff, Kismet, ethereal, and ettercap are employed which analyze the packets (or fragments), as a consequence, and approximately 192 times fragments are intercepted in the transmission. Thus, we can conclude that the DNP3 pseudo-transport layer has a lack of security design, or the DNP3 pseudo-transport layer was designed without considering any security. In [11,18,33], the number of attacks is defined and detected as part of the SCADA system, and security mechanisms are also used that protect the SCADA communication against several potential attacks and ensure the SCADA platform is invulnerable [12,13,25,27,29]. However, security is accounted in SCADA systems or/and SCADA protocols, with the exception of the deliberation of pseudo-transport layer security.

During the fragments interception shown in Figure 11 and Figure 12, the SCADA nodes are configured without any security paradigms such as firewalls, demilitarized zone (DMZ), antivirus protection, *etc.*, which determine the approximate security level during the transmission of fragmented payloads. However, if payload security was enhanced, the receiver would also be made aware of adversaries by contents verification. The fragmented payload hash digest is computed 200 times and transmitted along the original payload; upon receiving, the receiver also computes the hash digest of the original payload to compare with the sender hash digest. If two hash digest values are matched, then the receiver assumes that the payload came from an original source; otherwise, the payload contents are discarded and the exception (*i.e.*, payload contents have not been verified, there is chanced of adversary in transmission), is generated against the adversary.

In the existing studies [45,46,47,48], several limitations of SHA-2 hashing function are analyzed and creaking tools are employed; it is also assumed that the hash code is breakable. However, we did not fully succeed in breaking the computed hashing codes, or the results were captured with zero impact. In the case where the computed hashing values are breakable, we propose a method called a digital signature to resolve these issues. In this method, a fragmented payload hash digest is computed $H_{digest(S,Q)}^{TL}$ and a private key is deployed on the hash digital $Pr_{\left(k,S\right)}(\;H_{digest(S,Q)}^{TL}$), which acts as a digital signature. The original payload $Q_{\left(S,h,b\right)}^{TL}$ and digital signature $Pr_{\left(k,S\right)}(h_{digest(S,Q)}^{TL}$) are then encrypted with a public key $Pu_{\left(k,R\right)}$ of the receiver(R) as $Pu_{\left(k,R\right)}(Q_{\left(S,h,b\right)}^{TL},\;\;Pr_{\left(k,S\right)}(h_{digest(S,Q)}^{TL}$)), and transmitted to the remote side. Upon receiving, the receiver uses the sender (S) public key $Pu_{\left(k,S\right)}$ and the private key $Pr_{\left(k,R\right)}$ of the receiver(R) to open (or decrypt) the original payload $Q_{\left(S,h,b\right)}^{TL}$ and hash digest $H_{digest(S,Q)}^{TL}$. Subsequently, the hash digest of $Q_{\left(S,h,b\right)}^{TL}$ is calculated, and is designated as $H_{digest(R,Q)}^{TL}$ and compared with $H_{digest(S,Q)}^{TL}$. The keys such as private keys and public keys are defined and generated using an RSA algorithm; however, the keys are distributed statistically among the network nodes. As a consequence, we concluded that the hash digest is secured and the payload contents are not altered during transmission, even in cases of adversary. In study [49], the attack scenarios were conducted, in which the authentication and confidentiality attacks such as brute force, cryptography key cracking, eavesdropping, and man-in-the-middle are launched 200 times and the numbers of detected attacks are counted and visualized [11,49]. As a result, minimal impact is computed that is so far able to break the hash digest; also, it is very difficult for an adversary to inject, modify, and delete the sensitive information of the payload.

## 10. Multimedia Contexts

In this study, various multimedia contexts are employed in the form of text and images. The human machine interface (HMI) is designed and installed at both sides of the transmission. The basic configuration and setup, including the connection type (*i.e.*, TCP/IP), IP addresses, Port numbers, Channel setting, *etc.* required between the main controller and the remote controller are visualized as part of HMI. During transmission, the total number of bytes fragmented in the case of sending and responding (as part of the pseudo-transport layer), are also visualized which make it convenient for the end user prospective. The fragments flow in sequence and are shown on HMI at both sides of the transmission; the end users or operators can check the flow of fragments during the construction and distribution at both sides using sharing media (*i.e.*, team viewer *etc.*), which also determines the effects of networks adversaries, in the case where abnormal flows are visualized.

In a few cases, the main controller requires exceptional reports and screen shots (or images) of the physical setup, such as sensors, actuators, PLCs, and hardware devices; the information is then secured from the network adversaries, the images are transmitted in compressed form in order to minimize the memory space, and the security using the SHA-2 algorithm is deployed before responding to the main controller. Normally, SCADA systems are designed and used for low bandwidth; therefore, hashing is considered a secure and reliable approach [1,4]. In the case where there are potential adversaries that successfully break the hashing value, a digital signature is considered as the best approach according to the best of our knowledge and according to our measurements.

## 11. Significance of Study

Hashing is a good approach which verifies the payload contents by comparing the computed hashing values of the sender and receiver. In this study, pseudo-transport layer security issues are analyzed and the SHA-2 hashing algorithm is selected and deployed on the fragmented payload; TPDU is made up of user bytes and a header byte, while the FIR, FIN, and sequence number are part of TPCI. In the case where an adversary causes an interruption (*i.e.*, injection, modification, and deletion) by means of FIR, FIN, and sequence number, he/she cannot be successful because the hash digests are computed at both sides and compared at the remote side; if he/she is successful, the digital signature is computed for the hash value(s). As a consequence, the overall transmission is secured from the adversaries. This study also employed various multimedia contexts in the form of text and images, while security development and communication have been demonstrated to make information more convenient and reliable for the user.

## 12. Conclusions and Future Work

This study used a simulation based environment of water pumping system, and SCADA wireless sensors based network system to deploy the cryptography mechanism while communicating over unsecured network, or over Internet communication. Furthermore, the main security issues realized during the pseudo-transport layer disassembly and reassembling process are highlighted and a security solution using the SHA-2 hashing function is deployed, which ensures the integrity of bytes received/transmitted from/to the data link layer. Therefore, a DNP3 pseudo-transport layer stack has been designed and evaluated from formal evidence, security implementation is employed, and evidence is given of the protection against byte verification issues.

In future work, the SCADA system information will be accessed and monitored via cellular phones; and the SCADA/DNP3 testbed attack (abnormal) setup will be developed and simulation tools or software will be used to test the integrity attacks such as packet/data injection, packet/data replay, and data (byte) deletion and others. The security percentage will be measured based on the attack impact percentage on the overall system (or at the pseudo-transport layer stack). The other cryptography functions such as asymmetric and symmetric will also be deployed and security results will be validated against attacks such as shared key guessing, brute force, cracking key, man-in-the-middle, and others.

## Figures and Tables

**Table 1:** Attack taxonomy for the DNP3 pseudo-transport layer.

No.	Attacks	Attacks Instances (Description)
1	Passive Network Reconnaissance	Interception of main station, sub-controller, and network information.
2	Baseline Response Replay	Interruption, modification, and fabrication of the main station and sub-controller.
3	Rogue Interloper	Interruption, modification, and fabrication of the main station, sub-controller, and network information.
4	Fragment Interruption	Interruption of the main station and sub-controller.
5	Sequence Modification	Interception of Main Station, sub-controller, and network information.

**Table 2:** Attack taxonomy for the DNP3 data link layer.

No.	Attacks	Attacks Instances (Description)
1	Passive Network Reconnaissance	Interception of main station, sub-controller, and network information.
2	Baseline Response Replay	Interruption, modification, and fabrication of the main Station and sub-controller.
3	Rogue Interloper	Interruption, modification, and fabrication of the main station, sub-controller, and network information.
4	Length Overflow Attack	Interruption and modification of the main station and sub-controller.
5	Flag Attack	Interruption of sub-controller.
6	Reset Function Attack	Interruption and modification of the main station and sub-controller
7	Unavailable Function Attack	Interruption of the main station
8	Destination Address Alteration	Interruption, modification, and fabrication of the main station, sub-controller, and network information

**Table 3:** Security notations.

Notations	Description
$Q^{TL}\left(B\right)$	Assembled bytes.
$f_{Q}:b_{i}$	Disassembled bytes.
$f_{\alpha }:\left(Q_{h}^{TL}\right)$	Manipulated header bytes.
$f_{\alpha }:\left(Q_{b}^{TL}\right)$	Manipulated data bytes, after disassembling.
k → lim	K is dual integer that defines the limit (lim).
$\alpha_{TL}=\wedge$	User defined index pointer.
$f_{H}$	User defined hashing function.
$f_{Comp^{H}}$	Hashing comparison function.
$f_{w}:w_{h,b}$	User defined relation function.
$f_{p}:p_{h,b}$	User defined bytes separator function.
